# Locally aggressive monostotic fibrous dysplasia of the cervical spine mimicking malignancy: a case report and literature review

**DOI:** 10.1051/sicotj/2019024

**Published:** 2019-09-27

**Authors:** Audrey Milon, Marc Polivka, Fréderique Larousserie, Guillaume Lot, Jean-Marc Ziza, Jean-Denis Laredo

**Affiliations:** 1 Department of Radiology, Hôpital Lariboisière, Assistance Publique des Hôpitaux de Paris 2 rue Ambroise Paré 75010 Paris France; 2 Department of Pathology, Hôpital Lariboisière, Assistance Publique des Hôpitaux de Paris 2 rue Ambroise Paré 75010 Paris France; 3 Department of Pathology, Hôpital Cochin, Assistance Publique des Hôpitaux de Paris 27 rue du Faubourg Saint-Jacques 75014 Paris France; 4 Department of Neurosurgery, Fondation Ophtalmologique de Rothschild 29 rue Manin 75019 Paris France; 5 Department of Rheumatology, Hôpital La Croix Saint-Simon 125 rue d’Avron 75020 Paris France

**Keywords:** Monostotic fibrous dysplasia, Cervical spine, Pathological fracture, Aggressive

## Abstract

We report the case of a 30-year-old woman with histologically proven monostotic fibrous dysplasia of C2 revealed by a pathological fracture of the odontoid process. Radiological investigations showed a ground-glass mineralization of the vertebral body, a centimetric lytic area with poorly defined margins involving the inferior part of the vertebral body and inferior endplate and a fracture through an osteolytic area in the base of the odontoid process.

Owing to the vertebral instability, a surgical procedure combining C0–C5 fixation and posterior bone grafting was performed. The surgical biopsy was inconclusive and pathological confirmation was finally obtained through a percutaneous needle biopsy under fluoroscopic guidance. At 26-month follow-up, the patient still experienced mild persistent cervical posterior neck pain and stiffness possibly related to a C5–6 laxity below the intervertebral fixation.

This case combines three radiological findings, which are unusual in fibrous dysplasia: monostotic presentation involving the spine, some aggressive radiographic features, and a pathological fracture.

## Introduction

Fibrous dysplasia (FD) is a non-neoplastic tumor-like congenital process characterized by a localized defect in osteoblastic differentiation and maturation, with replacement of normal bone with large fibrous stroma and islands of immature woven bone. It is subclassified as monostotic and polyostotic lesions [1]. The spine is affected in only 2.5% of cases [2] and monostotic FD of the spine is uncommon. This case report covers the diagnosis and treatment of a patient with monostotic FD of C2 with a mixed osteosclerotic and osteolytic radiologic appearance, which was revealed by a pathological fracture of the odontoid process.

## Case report

**Figure 1:**
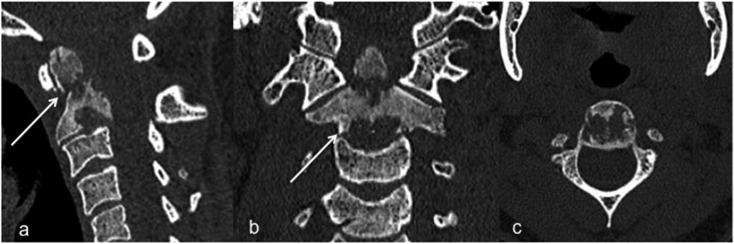
(a) Sagittal, (b) coronal and (c) axial CT-scan reformations of the upper cervical spine in a 30-year-old woman, showing the mixed appearance of the C2 vertebral body with a ground-glass appearance and osteolytic areas in the base of the odontoid process with a pathologic fracture (thin arrow) and in the inferior part of the vertebral body with partial destruction of the inferior endplate (thick arrow).

The patient was a 30-year-old woman who presented with a considerable spontaneous pain in the upper neck. She had no other significant medical history. Physical examination revealed a cervical stiffness without neurologic signs or symptoms. Radiographs of the cervical spine revealed a pathological fracture involving the odontoid process of C2. CT confirmed the fracture of the odontoid process and the replacement of normal bone by ground-glass mineralization and a centimetric lytic area with poorly defined margins involving the inferior part of the vertebral body and inferior endplate (Figure 1).

**Figure 2:**
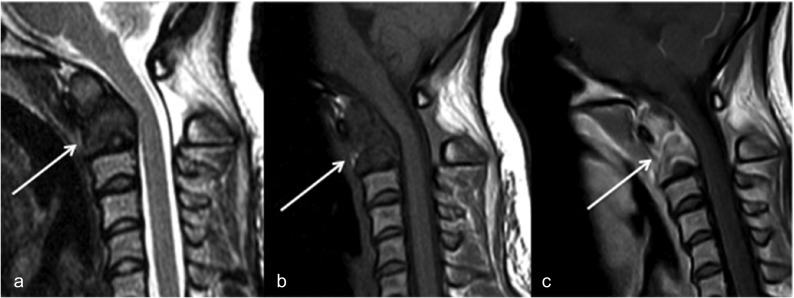
T2-weighted, T1-weighted, T1-weighted with intra-venous contrast administration midline sagittal MR images of the cervical spine showing the low signal intensity of the C2 vertebral body and odontoid process (a, b), with enhancement after contrast administration (c), and a solution at the junction between the odontoid process and vertebral body (a–c).

MRI of the cervical spine revealed low signal intensity of the whole C2 vertebra on both T1- and T2-weighted images, with mild homogenous enhancement after gadolinium administration that suggested a fibrous and/or mineralized content (Figure 2). There was no soft tissue extension and the C2–3 intervertebral disc was normal. Standard blood laboratory investigations, including C-reactive protein level, gave normal serum values. A full metastatic imaging workup including whole-body CT revealed no other abnormal findings.

**Figure 3:**
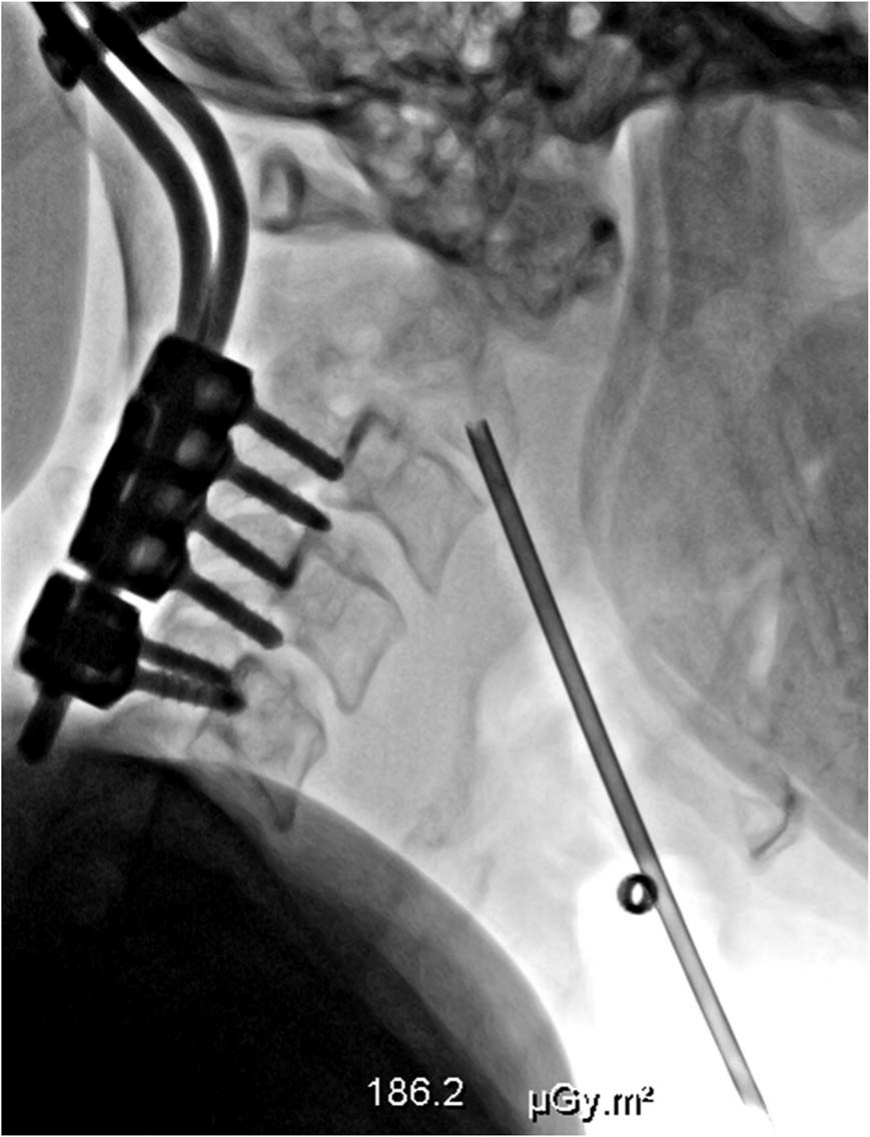
Percutaneous needle biopsy of C2 under fluoroscopic guidance with an anterolateral approach.

Owing to the odontoid process fracture and the vertebral instability, a surgical procedure combining C0–C5 fixation and posterior bone grafting was decided. The surgery was performed in the prone position and under general anesthesia. The neural arch of C2 was resected up to the lateral masses, and the bone resected was sent for pathological and bacteriological examinations. Screws (4.5-mm diameter, 12-mm size) were inserted in lateral masses of C3, C4, and C5 bilaterally according to the technique of Roy–Camille. Two vertical rods connected to an occipital plate were then fixed to C3, C4, and C5 screws and the plate was fixed to the occiput, thanks to three screws bilaterally. An autologous bone graft was withdrawn from the posterior iliac crest and placed from occiput to C3. Pathological examination of the resected bone was inconclusive as well as microbiology for Gram staining and bacteriological cultures including acid-fast bacilli and fungal assays. Therefore, a percutaneous needle biopsy of the osteolytic inferior part of the C2 vertebral body was performed under local anesthesia and fluoroscopic guidance. A 11-gauge 10-cm-long coaxial biopsy needle (KBC1110, Merit Medical^®^, Galway, Ireland) was advanced up to C2 vertebral body through an anterolateral ascending approach between the jugulo-carotid bundle and the trachea-oesophagus, and bone samples from C2 vertebral body were obtained (Figure 3). The final pathological diagnosis was FD, confirmed by a pathological review in a second institution. No secondary aneurysmal bone cyst and no sign of malignant transformation were seen. The patient received intravenous pamidronate (60 mg/day over 3 days every 6 months). At 3-month follow-up, the patient was doing well, except for mild cervical posterior neck pain and a follow-up radiograph showed that the fixation material was in good position. Some widening of the C5–6 facet joints, which could correspond to joint laxity below the fixed cervical levels, was noted. At 26-month follow-up, the patient still experienced mild persistent cervical posterior neck pain and stiffness, possibly related to the C5–6 laxity. Since the patient was pregnant at this last consultation, static and dynamic radiographic examinations as well as discussion of an indication of a C5–6 anterior joint fusion were postponed.

## Discussion

FD is a genetic, non-inherited condition with no sex predilection that results in a somatic mosaicism of affected osteoblastic cells producing a poorly organized fibrous connective tissue interwoven with trabeculae of immature bone [3]. It appears to be caused by mutations in the *GNAS1* gene that encodes for the alpha subunit of the stimulatory G-protein Gs [4].

We report a case of monostotic FD of C2 in a 30-year-old woman with a locally aggressive radiologic appearance, which was revealed by a pathological fracture of the odontoid process.

The spine accounts for only 1.4% of cases of monostotic FD [5]. Including ours, 18 cases of monostotic FD involving the cervical spine have been previously reported to our knowledge [6–20]. These 18 cases concerned 13 males and five females, most commonly in the third to fifth decades of life (mean age 32.5 years [range 11–56]), and involved C2 in seven cases (39%), C4 in five cases, C1 in three cases, and C3, C6, and C7 in one case each. Patients most commonly presented with axial neck or back pain, but in some cases, FD was an incidental finding. Only one patient had neurological symptoms, i.e. a 35-year-old man who presented a cervico-brachial neuralgia with sensitive symptoms.

Cervical FD may be revealed by a pathological fracture. Back to 1989, 11 cases including ours (mean age 37 years [range 17–63) of monostotic (*n* = 4) or polyostotic (*n* = 7) FD of the cervical spine revealed by a pathological fracture have been reported (Table 1). Interestingly, 5 out of the 11 cases involved C2. Only one of these was monostotic.

**Table 1:** Reported cases of cervical spine fibrous dysplasia presenting with a pathological fracture.

Report	Sex	Age	Neurological symptoms	Location	Monostotic/polyostotic	Treatment	Follow-up (month)
Our case	F	31	No	C2	Monostotic	Bone graft; posterior fixation	Asymptomatic at 18 months follow-up
Wu et al. [20]	M	48	No	C2–C3	Monostotic (non-segmentation of C2–C3)	Curettage; posterior fixation	Asymptomatic at 34-month follow-up
	M	28	No	C2	Monostotic	Curettage; anterior fixation	Asymptomatic at 33-month follow-up
	M	53	Yes	C7	Polyostotic	Excision; anterior and posterior fixation	Asymptomatic at 42-month follow-up
	M	17	Yes	C2	Polyostotic	Posterior fixation	Asymptomatic at 28-month follow-up
Lee et al. [34]	M	63	Yes	C4	Polyostotic	Corporectomy; allograft; posterior fixation	No follow-up
Dang et al. [35]	M	35	Yes	C2/C3	Polyostotic	Percutaneous vertebroplasty	Stable pain relief and neurologic improvement at 12-month follow-up
Marshmann et al. [13]	M	35	No	C3	Monostotic	Corpectomy; fixation	Asymptomatic at 18-month follow-up
Medow et al. [36]	F	40	No	C3	Polyostotic	Synthetic bone graft; posterior fixation	Asymptomatic at 25-month follow-up
Mezzadri et al. [37]	F	35	No	C5	Not stated	Corporectomy; synthetic graft; posterior fixation	Asymptomatic at 36-month follow-up
Stompro et al. [38]	M	26	Not stated	C2	Polyostotic	Immobilizer brace	No follow-up

CT features of vertebral FD do not differ from those of extraspinal location and included well-defined predominantly osteolytic lesions with ground-glass mineralization, sclerotic rim formation, an expansile nature with bone remodeling, and rarely cortical disruption. Vertebral body weight loss is frequent [21]. On T1-weighted images, low signal intensity is present in 67% of the lesions and heterogeneous signal intensity in 33%. On T2-weighted images, lesions show heterogeneous signal intensity with a low signal intensity rim in half of the cases; after intravenous gadolinium administration, lesion enhancement is homogeneous in 50% of cases and heterogeneous in 50% [21]. This diversity of the MR findings in FD is explained by their variable contents in bony trabeculae, cellularity, collagen fibers, cystic changes, and hemorrhage [22].

FD is traditionally considered to stop growing in the mature skeleton, but this may not actually be the case, especially with polyostotic presentation [22, 23]. In a single case of FD involving C2 in a 21-year-old man who underwent posterior spinal fusion from C1 to C3 performed with use of two cortico-cancellous grafts from the posterior iliac crest, the routine radiography follow-up 20 years later demonstrated extension of the expansile lesion through the bone graft to C3, with the classical ground-glass appearance. MRI confirmed the extension of the FD lesion into the posterior elements of C3 through the fusion bone graft [9, 24].

Our case exhibited some aggressive radiological features, namely a pathological fracture of the odontoid process and an osteolytic area involving the inferior part of the C2 vertebral body, which led us to perform a guided biopsy for pathological evaluation. A locally aggressive variant of FD characterized by cortical destruction that mimics malignancy on CT and MRI has been described [25, 26]. A large number of reported cases of locally aggressive FD involved the craniofacial skeleton, especially the maxilla and mandible in young patients [25, 27, 28]. Among the 14 cases of locally aggressive FD outside the skull previously reported, none was involving the spine [25, 26, 28–31].

Possible other causes of aggressive radiological patterns with cortical destruction and a soft tissue mass encountered in FD include malignant transformation and secondary aneurysmal bone cyst formation. Vertebral FD with secondary aneurysmal bone cyst formation is uncommon and usually manifests as an expanding lesion [32, 33]. Malignant transformation of vertebral FD is very uncommon and more frequent in polyostotic than monostotic FD [25]. Both secondary aneurysmal bone cyst formation and malignant transformation were absent in our case.

In conclusion, cervical FD may present in a young adult as a monostotic lesion, particularly in C2, with a pathological fracture and some aggressive radiologic features. In such cases, a ground-glass mineralization may suggest the diagnosis but a biopsy is required.
